# An Updated Meta-Analysis for Safety Evaluation of Alirocumab and Evolocumab as PCSK9 Inhibitors

**DOI:** 10.1155/2023/7362551

**Published:** 2023-01-04

**Authors:** Hye Duck Choi, Ji Hae Kim

**Affiliations:** College of Pharmacy, Yeungnam University, Gyeongbuk, Republic of Korea

## Abstract

**Background:**

Alirocumab and evolocumab, as protein convertase subtilisin kexin type 9 (PCSK9) inhibitors, have been reported to reduce cardiovascular risk. This meta-analysis is aimed at updating the safety data of PCSK9 inhibitors.

**Methods:**

We assessed the relative risk for all treatment-related adverse events, serious adverse events, diabetes-related adverse events, and neurocognitive and neurologic adverse events with PCSK9 inhibitors compared to controls (placebo or ezetimibe). In addition, we conducted a meta-analysis to quantitatively integrate and estimate the adverse event rates in long-term studies.

**Results:**

There were no significant differences between PCSK9 inhibitors and controls in the relative risk analysis. In a subgroup analysis of each PCSK9 inhibitor, alirocumab treatment significantly reduced the risk of serious adverse events compared to control treatment (risk ratio (RR) = 0.937; 95% confidence interval (CI), 0.896–0.980), but no significant difference was observed with evolocumab treatment (RR = 1.003; 95% CI, 0.963–1.054). Moreover, alirocumab treatment afforded a significant reduction in the risk of diabetes-related adverse events compared to control treatment (RR = 0.9137; 95% CI, 0.845–0.987). The overall incidence (event rate) of long-term adverse events was 75.1% (95% CI, 71.2%–78.7%), and the incidence of serious long-term event rate was 16.2% (95% CI, 11.6%–22.3%).

**Conclusions:**

We suggest that alirocumab and evolocumab are generally safe and well tolerated and that their addition to background lipid-lowering therapy is not associated with an increased risk of adverse events or toxicity.

## 1. Introduction

Alirocumab and evolocumab are fully human monoclonal antibodies against the protein convertase subtilisin kexin type 9 (PCSK9) and modulate the upregulation of recycling and expression of low-density lipoprotein cholesterol (LDL-C) receptors at the cell surface, and increase LDL-C clearance from circulation [1]. Both PCSK9 inhibitors were approved by the Food and Drug Administration in 2015 and are indicated for patients with established cardiovascular disease to reduce the risk of myocardial infarction, stroke, and coronary revascularization [2]. In addition, they are used as an adjunct to diet, alone or in combination with other LDL-C-lowering therapies, in patients with primary hyperlipidemia.

In randomized controlled trials, alirocumab and evolocumab have been reported to reduce the risk of recurrent cardiovascular disease in patients following an acute coronary event and secondary prevention populations when added to background statin therapy [3, 4]. In terms of safety, PCSK9 inhibitors are well tolerated and favorable. However, injection-related adverse events, such as injection-site reactions and “flu-like” symptoms after injections, may be a limitation in some patients [1]. In addition, long-term follow-up data on the efficacy or safety of PCSK9 inhibitors are insufficient, and some issues regarding their potential impact on neurocognitive- or diabetes-related risk have not been clearly uncovered [5].

A previous meta-analysis including 25 randomized controlled trials found that alirocumab and evolocumab are generally safe. However, it was reported that alirocumab increased the rate of injection-site reactions, while evolocumab reduced the rate of abnormal liver function [6]. In systematic reviews that evaluated concerns related to diabetes mellitus, the PCSK9 inhibitors were not associated with the risk of new-onset diabetes and adverse events of diabetes mellitus [7, 8]. Similarly, there was no increased risk of neurocognitive adverse events [9]. Since then, more clinical studies of both PCSK9 inhibitors have been reported.

This meta-analysis was conducted to update the safety data for PCSK9 inhibitors to assess the relative risk of alirocumab and evolocumab compared with placebo (or ezetimibe) and to quantitatively integrate and estimate the incidence of adverse events in long-term studies.

## 2. Methods

### 2.1. Search Strategy and Study Selection

We searched for published articles reporting adverse events associated with alirocumab and evolocumab in MEDLINE (OVID and PubMed), EMBASE, the Cochrane Library, and http://ClinialTrials.gov. The search was completed on October 30, 2021. The following search terms were used: *PCSK9 inhibitors, PCSK9 antibody, evolocumab, AMG 145, alirocumab, SAR236553,* and *REGN727*. We reviewed the reference lists of the retrieved articles and searched the relevant reviews to identify additional eligible studies. There were no restrictions on any publication.

Two authors independently reviewed and selected studies for inclusion in the systematic review. The inclusion criteria were as follows: (1) phase 2, 3, or 4 clinical trials; (2) administration of alirocumab or evolocumab; and (3) safety or adverse drug events. Disagreement about the inclusion of an article in the evaluation was resolved through discussion. For a clinical trial described in multiple reports, we extracted data from the most complete account and used the other publications only for clarification.

The study protocol for this meta-analysis was registered in the International Prospective Register for Systematic Reviews (PROSPERO) CRD42022328637.

### 2.2. Data Extraction and Quality Assessment

Two authors independently reviewed detailed full-text articles. The data were extracted from each study: number and characteristics of participants, treatment administered (dose regimen and periods), and adverse events. The bias risk of the included studies was assessed by two authors using the Cochrane RoB 2 criteria: bias arising from the randomization process, bias due to deviations from intended interventions, bias due to missing outcome data, bias in the measurement of the outcome, and bias in the selection of the reported result [10]. Disagreements between the two authors were resolved by consensus after discussion.

### 2.3. Meta-Analysis and Statistical Analysis

To evaluate treatment safety, we compared the total number of adverse events and serious adverse events reported in participants treated with alirocumab or evolocumab vs. those treated with placebo or ezetimibe. Moreover, we assessed the total number of diabetes-related, neurocognitive, and neurologic adverse events reported in both treatment groups. Studies with a follow-up period of at least 48 weeks were included to estimate the incidence of long-term adverse events.

The *χ*^2^ test (employing Q statistics) and the calculating *I*^2^ values were used to assess heterogeneity among including studies [11]. Based on the results of the heterogeneity test in each analysis, a fixed-effects model or a random-effects model was applied to the analysis [12, 13].

Publication bias was examined using Begg's method and Egger's regression test [14, 15]. Also, we performed sensitivity analyses by excluding the contribution of each study to the meta-analysis data in turn.

We performed all statistical analyses using the Comprehensive Meta-analysis Software version 2 (CMA 26526; Biostat, Englewood, NJ, USA). All *P* values were two-sided, and *P* values <0.05 was considered to indicate statistical significance.

## 3. Results

### 3.1. Study Characteristics and Risk of Bias Assessments

**Figure 1:**
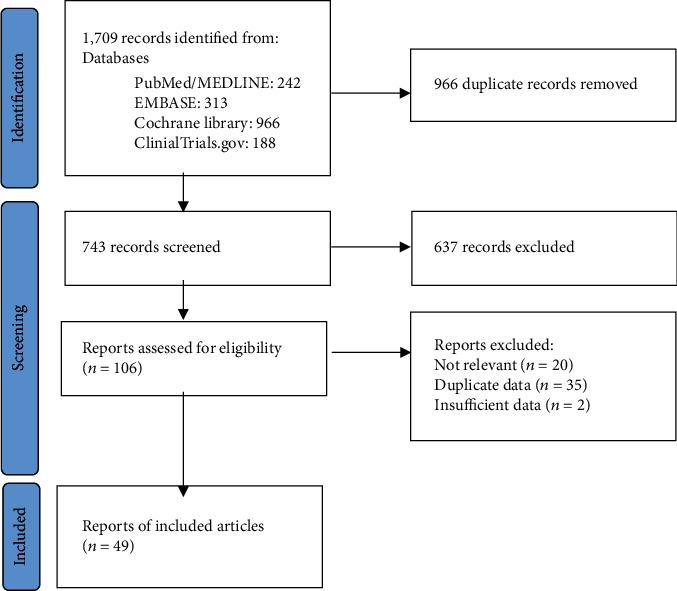
PRISMA flow diagram of the process for selection of relevant studies.

A total of 1,709 articles were identified in the literature search. The titles and abstracts of 743 articles were reviewed after excluding duplicates. Of these articles, 637 were excluded, and the full texts of 106 articles were assessed for meeting the eligibility criteria. A further 47 articles were excluded, and the data from the remaining 49 articles were finally included in the present meta-analysis (Figure 1). The general characteristics of included studies are shown in Table 1.

Risk of bias assessments for each study, including all domain judgments and support for judgment, are represented in the risk of bias section in Table 1. The risk of bias in outcomes across all studies was similar and predominately of ‘some concerns' (Supplementary Table S1).

### 3.2. Meta-Analysis of All Adverse Events and Serious Adverse Events

Forty-seven studies were included to evaluate any treatment-related adverse events. A total of 35,358 participants treated with PCSK9 inhibitors (alirocumab or evolocumab) and 30,710 participants treated with controls (placebo or ezetimibe) were assessed. No significant differences were observed between the two treatments (risk ratio (RR) = 1.023; 95% confidence interval (CI), 0.992–1.055) (Table 2).

**Figure 2:**
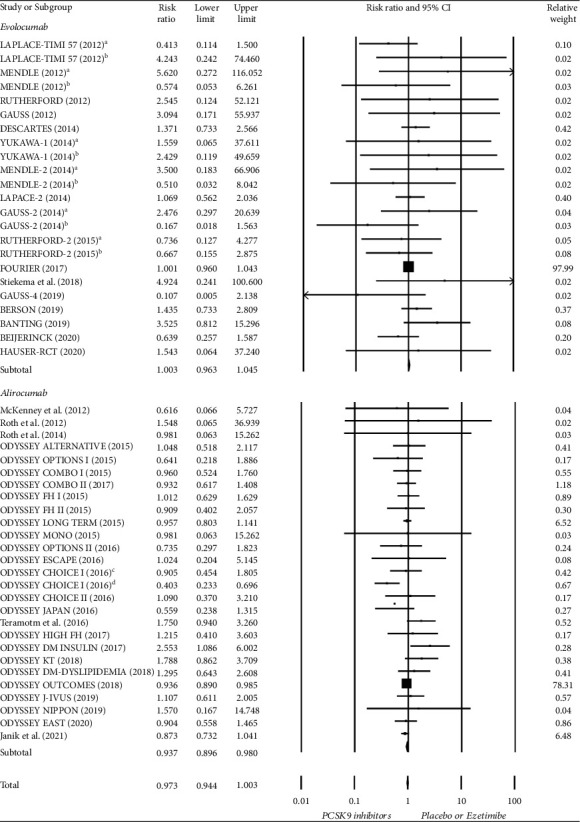
Forest plot of serious adverse events compared between PCSK9 inhibitors and control treatment (placebo or ezetimibe). ^a^ Treatment with evolocumab Q2W; ^b^ Treatment with evolocumab Q4W; ^c^ Treatment with alirocumab Q2W; ^d^ Treatment with alirocumab Q4W.

In the analysis of serious adverse events, 35,046 participants treated with PCSK9 inhibitors and 30,522 participants treated with controls from 44 studies were assessed. No significant differences were observed between the two treatments (RR = 0.973; 95% CI, 0.944–1.003). In the subgroup analysis of each PCSK9 inhibitor, alirocumab treatment significantly reduced the risk of serious adverse events compared to the control treatment, but no significant difference was observed with evolocumab treatment (alirocumab: RR = 0.937; 95% CI, 0.896–0.980; evolocumab: RR = 1.003; 95% CI, 0.963–1.054) (Figure 2).

### 3.3. Meta-Analysis of Diabetes-Related Adverse Events

**Figure 3:**
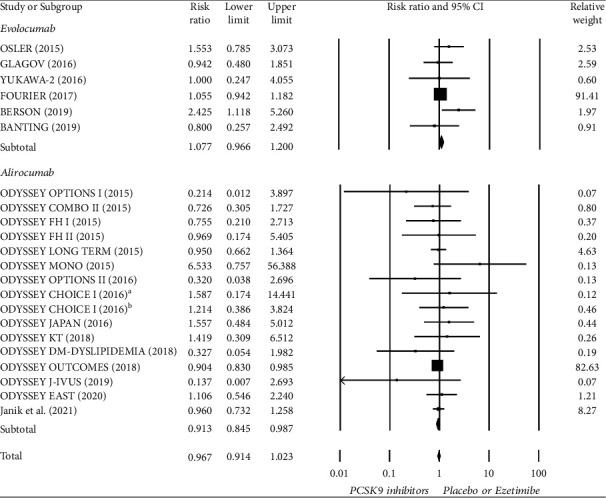
Forest plot of diabetes-related adverse events compared between PCSK9 inhibitors and control treatment (placebo or ezetimibe). ^a^ Treatment with alirocumab Q2W; ^b^ Treatment with alirocumab Q4W.

A total of 21 studies with 51,817 participants (27,770 treated with PCSK9 inhibitors and 24,047 treated with controls) were included. No significant difference was showed in the safety assessment of diabetes-related adverse events (RR = 0.967; 95% CI, 0.914–1.023). In subgroup analysis of each PCSK9 inhibitor, alirocumab treatment afforded a significant reduction in the risk of diabetes-related adverse events compared to control treatment (RR = 0.9137; 95% CI, 0.845–0.987) (Figure 3).

### 3.4. Meta-Analysis of Neurocognitive and Neurologic Adverse Events

**Figure 4:**
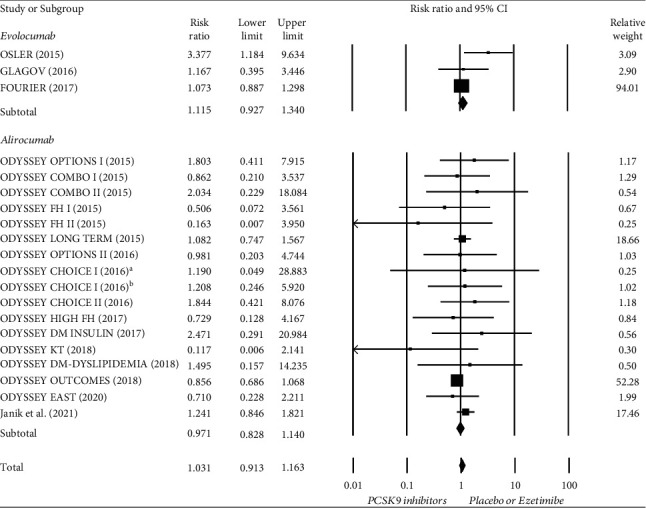
Forest plot of neurocognitive and neurologic adverse events compared between PCSK9 inhibitors and control treatment (placebo or ezetimibe). ^a^ Treatment with alirocumab Q2W; ^b^ Treatment with alirocumab Q4W.

Nineteen studies, including 32,916 participants treated with PCSK9 inhibitors and 29,166 participants treated with controls, were assessed. There was no significant difference in the safety assessment of neurocognitive and neurological adverse events between the two treatments (RR = 1.031; 95% CI, 0.913–1.163). There were no significant differences in the subgroup analysis of each PCSK9 inhibitor (Figure 4).

### 3.5. Incidence of Long-Term Adverse Events

A total of 13 studies were assessed for the long-term risk of all and serious adverse events in 20,969 participants treated with PCSK9 inhibitors. The overall incidence (event rate) of long-term adverse events was 75.1% (95% CI, 71.2%–78.7%), and the incidence of long-term serious event rate was 16.2% (95% CI, 11.6%–22.3%) using the random-effects model (Table 2).

Long-term risk of diabetes-related adverse events was assessed in 10 studies including 24,745 participants treated with PCSK9 inhibitors, and the incidence of diabetes-related adverse events was 4.50% (95% CI, 3.10%–6.50%), when applied the random-effects model (Table 2).

The long-term risk of neurocognitive and neurological adverse events was assessed in 12 studies, including 30,571 participants treated with PCSK9 inhibitors. The incidence of neurocognitive and neurologic adverse events was 1.70% (95% CI, 1.10%–2.70%), when applied the random-effects model (Table 2).

### 3.6. Publication Bias and Sensitivity Analyses

We evaluated the publication bias and the results of Begg's and Egger's tests are shown in Table 2. Sensitivity analysis was also performed by recalculating all findings after omitting the data from each study included in the meta-analysis. The results were not significantly altered throughout this process.

## 4. Discussion

We performed this meta-analysis to update the safety data for PCSK9 inhibitors to evaluate the relative risks of alirocumab and evolocumab compared to controls. In addition, we conducted a meta-analysis to quantitatively integrate and estimate the incidence of adverse events in long-term studies, which is a meaningful approach for the safety evaluation of PCSK9 inhibitors.

Based on the results of meta-analysis, we suggest that adding PCSK9 inhibitors to statins or other lipid-lowering therapies is not associated with an increased risk of adverse events or toxicity. That is, no significant differences were found in any of the comparisons analyzed, including serious adverse events, diabetes-related adverse events, or neurocognitive and neurological adverse events. Interestingly, alirocumab therapy seems to have a lower risk of diabetes and serious adverse events, which is consistent with a previous meta-analysis [7]. These results may be due to the unique characteristics of alirocumab or the effects of background lipid-lowering therapy.

In particular, diabetes mellitus is a cardiovascular risk and a significant adverse event of lipid-lowering therapies such as statins. Therefore, the use of PCSK9 inhibitors that do not increase the risk of diabetes is recommended. However, considering that most patients with dyslipidemia are treated with combination therapy, diabetes-related monitoring should not be excluded.

Previous studies have reported a higher incidence of neurocognitive events in patients receiving PCSK9 inhibitors than in those receiving standard therapy, but other clinical studies or systematic reviews did not show an increase in neurocognitive deficits in patients receiving these inhibitors [16, 17]. In addition, our meta-analysis showed results that were consistent with those described above. It is known that neither cholesterol nor PCSK9 can cross the blood-brain barrier under normal conditions, and alirocumab or evolocumab also cannot cross the blood-brain barrier [18, 19]. Therefore, we suggest that PCSK9 inhibitors do not cause or increase neurocognitive or neurological adverse events. However, cognitive problems in geriatric patients remain an important issue that requires close monitoring.

One meta-analysis reported that long-term treatment with alirocumab or evolocumab reduced LDL-C levels and improved cardiovascular outcomes while showing a similar safety profile to non-PCSK9 inhibitor therapy [20]. We performed the present meta-analysis to estimate the incidence of long-term adverse events. The overall incidence of long-term adverse events in PCSK9 inhibitor therapy is rather high at 75.1%, but it should be evaluated through (possibly indirect) comparison with the incidence of other comparative drugs. The incidence of diabetes-related adverse events, and neurocognitive and neurological adverse events was estimated to be approximately 4.50% and 1.7%, respectively.

In addition, a recent systematic review suggested that no major safety issues associated with PCSK9 inhibitors were observed, which is consistent with our results [21]. They also suggested that the use of PCSK9 inhibitors significantly reduced the risk of MI, ischemic stroke, and coronary revascularization in patients with dyslipidemia or atherosclerotic cardiovascular disease. These results, including our meta-analysis, are the evidences that support the role of PCSK9 inhibitors as treatments for dyslipidemia, and are expected to further increase their clinical use.

Our study had several limitations. First, we performed a meta-analysis based on previously reported articles which were not necessarily complete or accurate and the results may be partially different when applied to individual patients. Second, significant heterogeneity was present in the analyses, and dividing the studies into subgroups or performing a sensitivity analysis failed to identify the sources of heterogeneity. Despite these limitations, this meta-analysis is meaningful in that it provides clinical evidence for better pharmacotherapy in patients with dyslipidemia.

## 5. Conclusions

There were no significant differences between the PCSK9 inhibitors and controls, including serious adverse events, diabetes-related adverse events, or neurocognitive and neurological adverse events. PCKS9 inhibitors are relatively safe and well tolerated, and their addition to background lipid-lowering therapy is not associated with an increased risk of adverse events or toxicity.

## Figures and Tables

**Table 1:** General characteristics of included studies.

Study	Phase	Participants	Duration, (weeks)	Intervention, N	Control, N	Background LMT	Statin	Risk of bias
*Evolocumab*								
LAPLACE-TIMI 57 (2012) [22]	2	HC	12	70/104/140 mg Q2W, 236 280/350/420 mg Q4W, 238	Placebo Q2W,78 Placebo Q4W, 77	Statin with or w/o EZE	Both	Low risk
MENDEL (2012) [23]	2	HC	12	70/104/140 mg Q2W, 136 280/350/420 mg Q4W, 135	Placebo Q2W, 45 Placebo Q4W, 45 EZE, 45	No LMT	None	Low risk
RUTHERFORD (2012) [24]	2	He FH	12	350/420 mg Q4W, 111	Placebo, 56	Stable LMT	Both	Some concerns
GAUSS (2012) [25]	2	HC	12	280/350/420 mg Q4W, 95	Placebo/EZE, 32	Stable LMT	Both	Low risk
DESCARTES (2014) [26]	3	HC	52	420 mg Q4W, 599	Placebo, 302	Stable LMT	Both/none	Some concerns
YUKAWA-1 (2014) [27]	2	HC	12	70/140 mg Q2W, 101 280/420 mg QM, 104	Placebo Q2W. 52 Placebo QM, 50	Stable statin	Both	Some concerns
MENDEL-2 (2014) [28]	3	HC	12	140 mg Q2W, 153 420 mg Q4W, 153	Placebo Q2W, 76 Placebo Q4W, 78 EZE, 77	Stable LMT	NA	Some concerns
LAPACE-2 (2014) [29]	3	HC	12	140 mg Q2W or 420 mg Q4W, 1117	Placebo, 558 EZE, 221	Stable statin	Both	Some concerns
GAUSS-2 (2014) [30]	3	HC	12	140 mg Q2W, 103 420 mg Q4W, 102	EZE, 51	Stable LMT	NA	Some concerns
OSLER (2015) [16]	2/3	HC He FH	48-56	140 mg Q2W or 420 mg Q4W, 2976	Standard therapy, 1489	NA	NA	Some concerns
TESLA part B (2015) [31]	3	He FH	12	420 mg Q4W, 33	Placebo, 16	Stable LMT	Both	Low risk
RUTHERFORD-2 (2015) [32]	3	He FH	12	140 mg Q2W, 110 420 mg Q4W, 110	Placebo Q2W, 54 Placebo Q4W, 55	Stable LMT	NA	Low risk
GALGOV (2016) [33]	3	HC + CAD	76	420 mg Q4W, 484	Placebo, 484	Stable LMT	Both	Low risk
YUKAWA-2 (2016) [34]	3	HC	12	140 mg Q2W or 420 mg Q4W, 202	Placebo, 202	Stable statin	Both	Some concerns
FOURIER (2017) [4]	3	HC	113	140 mg Q2W or 420 mg Q4W, 13769	Placebo, 13756	NA	Both	Low risk
TAUSSIG (2017) [35]	3	Homozygous FH	48	420 mg Q4W, 106	None	NA	NA	Some concerns
Stiekema et al. [36]	3b	HC	16	420 mg Q4W, 65	Placebo, 64	Stable LMT	Both	Some concerns
GAUSS-4 (2020) [37]	3	HC	12+52	140 mg Q2W, 19 420 mg Q4W, 21	EZE, 21	Stable statin	Both	Some concerns
BERSON (2019) [38]	3	HC + DM	12	140 mg Q2W or 420 mg Q4W, 657	Placebo, 324	ATO 20 mg	Both	Low risk
BANTING (2019) [39]	3	HC + DM	12	420 mg Q4W, 280	Placebo, 141	NA	NA	Low risk
BEIJERINCK (2020) [40]	3	HC + HIV	24	420 mg Q4W, 307	Placebo, 157	Stable LMT	Both	Some concerns
HAUSER-RCT (2020) [41]	3	Pediatric FH	24	420 mg Q4W, 104	Placebo, 53	Stable LMT	Both	Some concerns
*Alirocumab*								
McKenney et al. [42]	2	HC	12	50/100/150 mg Q2W or 200/300 mg Q4W, 151	Placebo, 31	ATO 10/20/40 mg	Both	Low risk
Roth et al. [43]	2	HC	8	150 mg Q2W, 61	Placebo, 31	Stable statin	Both	Low risk
Stein et al. [44]	2	He FH	12	150 mg Q2W or 150/200/300 Q4W, 62	Placebo, 15	Stable statin	Both	Low risk
Roth et al. [45]	3	HC	24	75/150 mg Q2W, 52	EZE, 51	No LMT	None	Low risk
ODYSSEY ALTERNATIVE (2015) [46]	3	Statin-intolerant HC	24	75/150 mg Q2W, 126	EZE, 124	Stable statin	Both	Low risk
ODYSSEY OPTIONS I (2015) [47]	3	HC	24	75/150 mg Q2W, 104	EZE, 101	Stable statin	Both	Low risk
ODYSSEY COMBO I (2015) [48]	3	HC	52	75/150 mg Q2W, 207	Placebo, 107	Stable LMT	Both	Low risk
ODYSSEY FH I & FH II (2015) [49]	3	He FH	78	75/150 mg Q2W, 167	Placebo, 81	Stable LMT	Both	Some concerns
ODYSSEY LONG TERM (2015) [50]	3	HC	78	150 mg Q2W, 1550	Placebo, 788	Stable LMT	Both	Low risk
ODYSSEY MONO (2015) [17]	3	HC	32	75/150 mg Q2W, 52	EZE, 51	No LMT	None	Some concerns
ODYSSEY OPTIONS II (2016) [51]	3	HC	2	75/150 mg Q2W, 103	EZE, 101	Rosuvastatin	Both	Low risk
ODYSSEY ESCAPE (2016) [52]	3	HC	18	150 mg Q2W, 41	Placebo, 21	NA	NA	Some concerns
ODYSSEY CHOICE I (2016) [53]	3	HC	48	75/150 mg Q2W,	Placebo/EZE	Stable LMT	Both/none	Low risk
ODYSSEY CHOICE II (2016) [54]	3	HC	24	75/150 mg Q2W or 300 mg Q4W, 573	Placebo, 229	Fenofibrate, EZE or diet	None	Some concerns
ODYSSEY JAPAN (2016) [55]	3	He FH	52	75/150 mg Q2W, 143	Placebo, 72	Stable LMT	Both	Low risk
Teramoto et al. [56]	2	HC	12	75/150 mg Q2W, 107	Placebo, 56	Stable LMT	Both	Some concerns
ODYSSEY COMBO II (2017) [57]	3	HC + ASCVD	104	75/150 mg Q2W, 411	EZE, 209	Stable LMT	Both/none	Low risk
ODYSSEY HIGH FH (2016) [58]	3	He FH	78	150 mg Q2W, 72	Placebo, 35	Stable LMT	Both	Some concerns
ODYSSEY DM INSULIN (2017) [59]	3	HC + type 2 DM	24	75/150 mg Q2W, 344	Placebo, 170	Stable LMT	Both	Some concerns
ODYSSEY KT (2018) [60]	3	HC	24	75/150 mg Q2W, 97	Placebo, 102	Stable LMT	Both	Some concerns
ODYSSEY DM-DYSLIPIDEMIA (2018) [61]	3b/4	HC + type 2 DM	24	75/150 mg Q2W, 275	Usual care, 137	Maximally tolerated dose of stain	Both	Some concerns
ODYSSEY OUTCOMES (2018) [3]	3	HC + ASC	257	75/150 mg Q2W, 9451	Placebo, 9443	Stable LMT	Both	Low risk
ODYSSEY J-IVUS (2019) [62]	4	HC + ASC	36	75/150 mg Q2W, 103	Standard therapy,	Stable LMT	Both	Some concerns
ODYSSEY NIPPON (2019) [63]	3	He FH Non FH	64	150 mg Q2W, 158	None	ATO 5 mg or nonstatin	Both	Some concerns
ODYSSEY HoFH (2020) [64]	3	Homozygous FH	24	150 mg Q2W, 45	Placebo, 24	Statin with or w/o EZE	Both	Some concerns
ODYSSEY EAST(2020) [65]	3	HC	24	75/150 mg Q2W, 406	EZE, 206	Maximally tolerated dose of stain	Both	Some concerns
Janik et al. [66]	4	He FH Non FH	96	75/150 mg Q2W, 1087	Placebo, 1084	Stable LMT	Both	Some concerns

Abbreviations: ATO: atorvastatin; ACS: acute coronary syndrome; ASCVD: atherosclerotic cardiovascular disease; CAD: coronary artery disease; DM: diabetes mellitus; EZE: ezetimibe; He FH: heterozygous familial hyperlipidemia; LMT: lipid modifying therapy; NA: not reported.

**Table 2:** Test of heterogeneity and publication bias.

		Test of heterogeneity			Publication bias	
	No. of study	*Q* value	*P* value	*I* ^2^	*P* value (Begg's)	*P* value (Egger's)
*Risk ratio*						
All adverse events	47	205.9	<0.001	74.26	0.357	0.007
Serious adverse events	44	50.37	0.419	2.720	0.181	0.230
Diabetes-related adverse events	20	21.64	0.420	2.961	0.155	0.311
Neurocognitive and neurologic adverse events	19	15.72	0.676	<0.001	0.103	0.341
*Event rate*						
Long-term all adverse events	13	227.7	<0.001	94.73	0.427	0.350
Long-term serious adverse events	13	583.6	<0.001	97.94	0.251	0.062
Long-term diabetes-related adverse events	10	353.4	<0.001	97.45	0.237	0.013
Long-term neurocognitive and neurologic adverse events	12	182.4	<0.001	93.97	0.269	0.409

## Data Availability

The data supporting this meta-analysis are from previously reported studies and datasets, which have been cited. The processed data are available from the corresponding author upon request.

## Abbreviations

CI: Confidence interval
LDL-C: Low-density lipoprotein cholesterol
PCSK9: Protein convertase subtilisin kexin type 9
RR: Risk ratio.

## Supplementary Materials

Supplementary 1 Table S1: Risk of bias.

Supplementary 2 Table S2: PRISMA 2020 checklist.

## References

[1] Rosenson R. S., Hegele R. A., Fazio S., Cannon C. P. (2018). The evolving future of PCSK9 inhibitors. *Journal of the American College of Cardiology*.

[2] Lloyd-Jones D. M., Morris P. B., Ballantyne C. M. (2017). 2017 focused update of the 2016 ACC expert consensus decision pathway on the role of non-statin therapies for LDL-cholesterol lowering in the management of atherosclerotic cardiovascular disease risk: a report of the American College of Cardiology Task Force on expert consensus decision pathways. *Journal of the American College of Cardiology*.

[3] Schwartz G. G., Steg P. G., Szarek M. (2018). Alirocumab and cardiovascular outcomes after acute coronary syndrome. *The New England Journal of Medicine*.

[4] Sabatine M. S., Giugliano R. P., Keech A. C. (2017). Evolocumab and clinical outcomes in patients with cardiovascular disease. *The New England Journal of Medicine*.

[5] Kosmas C. E., Skavdis A., Sourlas A. (2020). Safety and tolerability of PCSK9 inhibitors: current insights. *Clinical Pharmacology: Advances and Applications*.

[6] Zhang X. L., Zhu Q. Q., Zhu L. (2015). Safety and efficacy of anti-PCSK9 antibodies: a meta-analysis of 25 randomized, controlled trials. *BMC Medicine*.

[7] Chen TrQ W. G., Li C., Qin X., Liu R., Zhang M. (2020). Safety of Proprotein convertase Subtilisin/Kexin type 9 monoclonal antibodies in regard to diabetes mellitus: a systematic review and meta-analysis of randomized controlled trials. *American Journal of Cardiovascular Drugs*.

[8] de Carvalho L. S. F., Campos A. M., Sposito A. C. (2018). Proprotein convertase Subtilisin/Kexin type 9 (PCSK9) inhibitors and incident type 2 diabetes: a systematic review and meta-analysis with over 96,000 patient-years. *Diabetes Care*.

[9] Bajaj N. S., Patel N., Kalra R. (2018). Neurological effects of proprotein convertase subtilisin/kexin type 9 inhibitors: direct comparisons. *European Heart Journal - Quality of Care and Clinical Outcomes*.

[10] Higgins J. P. T., Thomas J., Chandler J. (2019). *Cochrane Handbook for Systematic Reviews of Interventions*.

[11] Cochran W. G. (1954). The combination of estimates from different experiments. *Biometrics*.

[12] Mantel N., Haenszel W. (1959). Statistical aspects of the analysis of data from retrospective studies of disease. *Journal of the National Cancer Institute*.

[13] DerSimonian R., Larid N. (1986). Meta-analysis in clinical trials. *Controlled Clinical Trials*.

[14] Begg C. B., Mazumdar M. (1994). Operating characteristics of a rank correlation test for publication bias. *Biometircs*.

[15] Egger M., Smith G. D., Schneider M., Minder C. (1997). Bias in meta-analysis detected by a simple, graphical test. *BMJ*.

[16] Koren M. J., Giugliano R. P., Raal F. J. (2014). Efficacy and safety of longer-term administration of evolocumab (AMG 145) in patients with hypercholesterolemia: 52-week results from the open-label study of long-term evaluation against LDL-C (OSLER) randomized trial. *Circulation*.

[17] Roth E. M., McKenney J. M. (2015). ODYSSEY MONO: effect of alirocumab 75 mg subcutaneously every 2 weeks as monotherapy versus ezetimibe over 24 weeks. *Future Cardiology*.

[18] O’Connell E. M., Lohoff F. W. (2020). Proprotein convertase subtilisin/kexin type 9 (PCSK9) in the brain and relevance for neuropsychiatric disorders. *Frontiers in Neuroscience*.

[19] Bandyopadhyay D., Ashish K., Hajra A., Qureshi A., Ghosh R. K. (2018). Cardiovascular outcomes of PCSK9 inhibitors: with special emphasis on its effect beyond LDL-cholesterol lowering. *Journal of Lipids*.

[20] Bai J., Gong L. L., Li Q. F., Wang Z. H. (2018). Long-term efficacy and safety of proprotein convertase subtilisin/kexin 9 monoclonal antibodies: a meta-analysis of 11 randomized controlled trials. *Journal of Clinical Lipidology*.

[21] Guedeney P., Giustino G., Sorrentino S. (2019). Efficacy and safety of alirocumab and evolocumab: a systematic review and meta-analysis of randomized controlled trials. *European Heart Journal*.

[22] Giugliano R. P., Desai N. R., Kohli P. (2012). Efficacy, safety, and tolerability of a monoclonal antibody to proprotein convertase subtilisin/kexin type 9 in combination with a statin in patients with hypercholesterolaemia (LAPLACE-TIMI 57): a randomised, placebo- controlled, dose-ranging, phase 2 study. *Lancet*.

[23] Koren M. J., Scott R., Kim J. B. (2012). Efficacy, safety, and tolerability of a monoclonal antibody to proprotein convertase subtilisin/kexin type 9 as monotherapy in patients with hypercholesterolaemia (MENDEL): a randomised, double-blind, placebo- controlled, phase 2 study. *Lancet*.

[24] Raal F., Scott R., Somaratne R. (2012). Low-density lipoprotein cholesterol-lowering effects of AMG 145, a monoclonal antibody to proprotein convertase subtilisin/kexin type 9 serine protease in patients with heterozygous familial hypercholesterolemia: the reduction of LDL-C with PCSK9 inhibition in heterozygous familial hypercholesterolemia disorder (RUTHERFORD) randomized trial. *Circulation*.

[25] Sullivan D., Olsson A. G., Scott R. (2012). Effect of a monoclonal antibody to PCSK9 on low-density lipoprotein cholesterol levels in statin-intolerant patients. *Journal of the American Medical Association*.

[26] Blom D. J., Hala T., Bolognese M. (2014). A 52-week placebo-controlled trial of evolocumab in hyperlipidemia. *The New England Journal of Medicine*.

[27] Hirayama A., Honarpour N., Yoshida M. (2014). Effects of evolocumab (AMG 145), a monoclonal antibody to PCSK9, in hypercholesterolemic, statin-treated japanese patients at high cardiovascular risk--primary results from the phase 2 YUKAWA study. *Circulation Journal*.

[28] Koren M. J., Lundqvist P., Bolognese M. (2014). Anti-PCSK9 monotherapy for hypercholesterolemia: the MENDEL-2 randomized, controlled phase III clinical trial of evolocumab. *Journal of the American College of Cardiology*.

[29] Robinson J. G., Nedergaard B. S., Rogers W. J. (2014). Effect of evolocumab or ezetimibe added to moderate- or high-intensity statin therapy on LDL-C lowering in patients with Hypercholesterolemia. *Journal of the American Medical Association*.

[30] Stroes E., Colquhoun D., Sullivan D. (2014). Anti-PCSK9 antibody effectively lowers cholesterol in patients with statin intolerance: the GAUSS-2 randomized, placebo-controlled phase 3 clinical trial of evolocumab. *Journal of the American College of Cardiology*.

[31] Raal F. J., Honarpour N., Blom D. J. (2015). Inhibition of PCSK9 with evolocumab in homozygous familial hypercholesterolaemia (TESLA Part B): a randomised, double-blind, placebo- controlled trial. *Lancet*.

[32] Raal F. J., Stein E. A., Dufour R. (2015). PCSK9 inhibition with evolocumab (AMG 145) in heterozygous familial hypercholesterolaemia (RUTHERFORD-2): a randomised, double-blind, placebo- controlled trial. *Lancet*.

[33] Nicholls S. J., Puri R., Anderson T. (2016). Effect of Evolocumab on progression of coronary disease in statin-treated patients: the GLAGOV randomized clinical trial. *Journal of the American Medical Association*.

[34] Kiyosue A., Honarpour N., Kurtz C., Xue A., Wasserman S. M., Hirayama A. (2016). A phase 3 study of Evolocumab (AMG 145) in statin-treated Japanese patients at high cardiovascular risk. *The American Journal of Cardiology*.

[35] Raal F. J., Hovingh G. K., Blom D. (2017). Long-term treatment with evolocumab added to conventional drug therapy, with or without apheresis, in patients with homozygous familial hypercholesterolaemia: an interim subset analysis of the open-label TAUSSIG study. *The Lancet Diabetes and Endocrinology*.

[36] Stiekema L. C. A., Stroes E. S. G., Verweij S. L. (2019). Persistent arterial wall inflammation in patients with elevated lipoprotein (a) despite strong low-density lipoprotein cholesterol reduction by proprotein convertase subtilisin/kexin type 9 antibody treatment. *European Heart Journal*.

[37] Koba S., Inoue I., Cyrille M. (2020). Evolocumab vs. ezetimibe in statin-intolerant hyperlipidemic Japanese patients: phase 3 gauss-4 trial. *Journal of Atherosclerosis and Thrombosis*.

[38] Lorenzatti A. J., Eliaschewitz F. G., Chen Y. (2019). Randomised study of evolocumab in patients with type 2 diabetes and dyslipidaemia on background statin: primary results of the BERSON clinical trial. *Diabetes, Obesity & Metabolism*.

[39] Rosenson R. S., Daviglus M. L., Handelsman Y. (2019). Efficacy and safety of evolocumab in individuals with type 2 diabetes mellitus: primary results of the randomised controlled BANTING study. *Diabetologia*.

[40] Boccara F., Kumar P. N., Caramelli B. (2020). Evolocumab in HIV-infected patients with dyslipidemia: primary results of the randomized, double-blind BEIJERINCK study. *Journal of the American College of Cardiology*.

[41] Santos R. D., Ruzza A., Hovingh G. K. (2020). Evolocumab in pediatric heterozygous familial hypercholesterolemia. *The New England Journal of Medicine*.

[42] McKenney J. M., Koren M. J., Kereiakes D. J., Hanotin C., Ferrand A. C., Stein E. A. (2012). Safety and efficacy of a monoclonal antibody to proprotein convertase subtilisin/kexin type 9 serine protease, SAR236553/REGN727, in patients with primary hypercholesterolemia receiving ongoing stable atorvastatin therapy. *Journal of the American College of Cardiology*.

[43] Roth E. M., McKenney J. M., Hanotin C., Asset G., Stein E. A. (2012). Atorvastatin with or without an antibody to PCSK9 in primary hypercholesterolemia. *The New England Journal of Medicine*.

[44] Stein E. A., Honarpour N., Wasserman S. M., Xu F., Scott R., Raal F. J. (2013). Effect of the proprotein convertase subtilisin/kexin 9 monoclonal antibody, AMG 145, in homozygous familial hypercholesterolemia. *Circulation*.

[45] Roth E. M., Taskinen M. R., Ginsberg H. N. (2014). Monotherapy with the PCSK9 inhibitor alirocumab versus ezetimibe in patients with hypercholesterolemia: results of a 24 week, double-blind, randomized phase 3 trial. *International Journal of Cardiology*.

[46] Moriarty P. M., Thompson P. D., Cannon C. P. (2015). Efficacy and safety of alirocumab vs ezetimibe in statin-intolerant patients, with a statin rechallenge arm: the ODYSSEY ALTERNATIVE randomized trial. *Journal of Clinical Lipidology*.

[47] Bays H., Gaudet D., Weiss R. (2015). Alirocumab as add-on to atorvastatin versus other lipid treatment strategies: ODYSSEY OPTIONS I randomized trial. *The Journal of Clinical Endocrinology and Metabolism*.

[48] Kereiakes D. J., Robinson J. G., Cannon C. P. (2015). Efficacy and safety of the proprotein convertase subtilisin/kexin type 9 inhibitor alirocumab among high cardiovascular risk patients on maximally tolerated statin therapy: the ODYSSEY COMBO I study. *American Heart Journal*.

[49] Kastelein J. J., Ginsberg H. N., Langslet G. (2015). ODYSSEY FH I and FH II: 78 week results with alirocumab treatment in 735 patients with heterozygous familial hypercholesterolaemia. *European Heart Journal*.

[50] Dufour R., Hovingh G. K., Guyton J. R. (2019). Individualized low-density lipoprotein cholesterol reduction with alirocumab titration strategy in heterozygous familial hypercholesterolemia: results from an open-label extension of the ODYSSEY LONG TERM trial. *Journal of Clinical Lipidology*.

[51] Farnier M., Jones P., Severance R. (2016). Efficacy and safety of adding alirocumab to rosuvastatin versus adding ezetimibe or doubling the rosuvastatin dose in high cardiovascular-risk patients: the ODYSSEY OPTIONS II randomized trial. *Atherosclerosis*.

[52] Moriarty P. M., Parhofer K. G., Babirak S. P. (2016). Alirocumab in patients with heterozygous familial hypercholesterolaemia undergoing lipoprotein apheresis: the ODYSSEY ESCAPE trial. *European Heart Journal*.

[53] Roth E. M., Moriarty P. M., Bergeron J. (2016). A phase III randomized trial evaluating alirocumab 300 mg every 4 weeks as monotherapy or add-on to statin: ODYSSEY CHOICE I. *Atherosclerosis*.

[54] Stroes E., Guyton J. R., Lepor N. (2016). Efficacy and safety of alirocumab 150 mg every 4 weeks in patients with hypercholesterolemia not on statin therapy: the ODYSSEY CHOICE II study. *Journal of the American Heart Association*.

[55] Teramoto T., Kobayashi M., Tasaki H. (2016). Efficacy and safety of alirocumab in Japanese patients with heterozygous familial hypercholesterolemia or at high cardiovascular risk with hypercholesterolemia not adequately controlled with statins - ODYSSEY JAPAN randomized controlled trial. *Circulation Journal*.

[56] Teramoto T., Kobayashi M., Uno K. (2016). Efficacy and safety of Alirocumab in Japanese subjects (phase 1 and 2 studies). *The American Journal of Cardiology*.

[57] Cannon C. P., Cariou B., Blom D. (2015). Efficacy and safety of alirocumab in high cardiovascular risk patients with inadequately controlled hypercholesterolaemia on maximally tolerated doses of statins: the ODYSSEY COMBO II randomized controlled trial. *European Heart Journal*.

[58] Ginsberg H. N., Rader D. J., Raal F. J. (2016). Efficacy and safety of Alirocumab in patients with heterozygous familial hypercholesterolemia and LDL-C of 160 mg/dl or higher. *Cardiovascular Drugs and Therapy*.

[59] Leiter L. A., Cariou B., Müller-Wieland D. (2017). Efficacy and safety of alirocumab in insulin-treated individuals with type 1 or type 2 diabetes and high cardiovascular risk: the ODYSSEY DM-INSULIN randomized trial. *Diabetes, Obesity & Metabolism*.

[60] Koh K. K., Nam C. W., Chao T. H. (2018). A randomized trial evaluating the efficacy and safety of alirocumab in South Korea and Taiwan (ODYSSEY KT). *Journal of Clinical Lipidology*.

[61] Ray K. K., Leiter L. A., Müller-Wieland D. (2018). Alirocumab vs usual lipid-lowering care as add-on to statin therapy in individuals with type 2 diabetes and mixed dyslipidaemia: the ODYSSEY DM- DYSLIPIDEMIA randomized trial. *Diabetes, Obesity & Metabolism*.

[62] Ako J., Hibi K., Tsujita K. (2019). Effect of alirocumab on coronary atheroma volume in japanese patients with acute coronary syndrome - the ODYSSEY J-IVUS trial. *Circulation Journal*.

[63] Teramoto T., Kiyosue A., Ishigaki Y. (2019). Efficacy and safety of alirocumab 150 mg every 4 weeks in hypercholesterolemic patients on non-statin lipid-lowering therapy or lowest strength dose of statin: ODYSSEY NIPPON. *Journal of Cardiology*.

[64] Blom D. J., Harada-Shiba M., Rubba P. (2020). Efficacy and safety of alirocumab in adults with homozygous familial hypercholesterolemia: the ODYSSEY HoFH trial. *Journal of the American College of Cardiology*.

[65] Han Y., Chen J., Chopra V. (2020). ODYSSEY EAST: alirocumab efficacy and safety vs ezetimibe in high cardiovascular risk patients with hypercholesterolemia and on maximally tolerated statin in China, India, and Thailand. *Journal of Clinical Lipidology*.

[66] Janik M. J., Urbach D. V., van Nieuwenhuizen E. (2021). Alirocumab treatment and neurocognitive function according to the CANTAB scale in patients at increased cardiovascular risk: a prospective, randomized, placebo-controlled study. *Atherosclerosis*.

