# Supplementary material for: An Updated Meta-Analysis for Safety Evaluation of Alirocumab and Evolocumab as PCSK9 Inhibitors
Source: Cardiovasc Ther. 2023 Jan 4;2023:7362551. doi: 10.1155/2023/7362551 (PMC9834631; doi:10.1155/2023/7362551)
Supplement: Supplementary 1 — Table S1: Risk of bias. [file 7362551.f1.pdf]

Supplementary Table 1. Risk of bias

Study	Randomisation process	Deviations from intended interventions	Missing outcome data	Measurement of the outcome	Selection of the reported results
LAPLACETIMI (2012)	Low risk	Low risk	Low risk	Low risk	Low risk
MENDLE (2012)	Low risk	Low risk	Low risk	Low risk	Low risk
RUTHERFORD (2012)	Low risk	Some concerns	Low risk	Low risk	Low risk
GAUSS (2012)	Low risk	Low risk	Low risk	Low risk	Low risk
DESCARTES (2014)	Some concerns	Low risk	Low risk	Low risk	Low risk
YUKAWA-1 (2014)	Some concerns	Low risk	Low risk	Low risk	Low risk
MENDLE-2 (2014)	Some concerns	Low risk	Low risk	Low risk	Low risk
LAPACE-2 (2014)	Some concerns	Some concerns	Low risk	Some concerns	Low risk
GAUSS-2 (2014)	Some concerns	Low risk	Low risk	Low risk	Low risk
OSLER (2015)	Low risk	Some concerns	Low risk	Some concerns	Low risk
TESLA Part B (2015)	Low risk	Low risk	Low risk	Low risk	Low risk
RUTHERFORD-2 (2015)	Low risk	Low risk	Low risk	Low risk	Low risk
YUKAWA-2 (2016)	Some concerns	Low risk	Low risk	Low risk	Low risk
GALGOV (2016)	Low risk	Low risk	Low risk	Low risk	Low risk
FOURIER (2017)	Low risk	Low risk	Low risk	Low risk	Low risk
TAUSSIG (2017)	NA	NA	Some concerns	NA	Low risk
Stiekema et al. (2018)	Low risk	Some concerns	Low risk	Some concerns	Low risk
GAUSS-4 (2019)	Low risk	Some concerns	Low risk	Some concerns	Low risk
BERSON (2019)	Low risk	Low risk	Low risk	Low risk	Low risk
BANTING (2019)	Low risk	Low risk	Low risk	Low risk	Low risk
BEIJERINCK (2020)	Some concerns	Some concerns	Low risk	Some concerns	Low risk
HAUSER-RCT (2020)	Some concerns	Some concerns	Low risk	Some concerns	Low risk
McKenney et al. (2012)	Low risk	Low risk	Low risk	Low risk	Low risk
Roth et al. (2012)	Low risk	Low risk	Low risk	Low risk	Low risk
Stein et al. (2012)	Low risk	Low risk	Low risk	Low risk	Low risk
Roth et al. (2014)	Low risk	Low risk	Low risk	Low risk	Low risk
ODYSSEY ALTERNATIVE (2015)	Low risk	Low risk	Low risk	Low risk	Low risk
ODYSSEY OPTIONS I (2015)	Low risk	Low risk	Low risk	Low risk	Low risk

ODYSSEY COMBO I (2015)	Low risk	Low risk	Low risk	Low risk	Low risk
ODYSSEY FH I, FH II (2015)	Low risk	Some concerns	Low risk	Low risk	Low risk
ODYSSEY LONG TERM (2015)	Low risk	Low risk	Low risk	Low risk	Low risk
ODYSSEY MONO (2015)	Some concerns	Low risk	Low risk	Low risk	Low risk
ODYSSEY OPTIONS II (2016)	Low risk	Low risk	Low risk	Low risk	Low risk
ODYSSEY ESCAPE (2016)	Low risk	Low risk	Some concerns	Low risk	Low risk
ODYSSEY CHOICE I (2016)	Low risk	Low risk	Low risk	Low risk	Low risk
ODYSSEY CHOICE II (2016)	Some concerns	Low risk	Low risk	Low risk	Low risk
ODYSSEY JAPAN (2016)	Low risk	Low risk	Low risk	Low risk	Low risk
Teramotm et al. (2016)	Low risk	Some concerns	Some concerns	Low risk	Low risk
ODYSSEY COMBO II (2017)	Low risk	Low risk	Low risk	Low risk	Low risk
ODYSSEY HIGH FH (2017)	Low risk	Some concerns	Low risk	Low risk	Low risk
ODYSSEY DM INSULIN (2017)	Some concerns	Low risk	Low risk	Low risk	Low risk
ODYSSEY KT (2018)	Low risk	Low risk	Low risk	Some concerns	Low risk
ODYSSEY DM-DYSLIPIDEMIA (2018)	Low risk	Some concerns	Low risk	Low risk	Low risk
ODYSSEY OUTCOMES (2018)	Low risk	Low risk	Low risk	Low risk	Low risk
ODYSSEY J-IVUS (2019)	Low risk	Some concerns	Some concerns	Some concerns	Low risk
ODYSSEY NIPPON (2019)	Some concerns	Low risk	Low risk	Low risk	Low risk
ODYSSEY HoFH (2020)	Low risk	Low risk	Some concerns	Low risk	Low risk
ODYSSEY EAST(2020)	Some concerns	Low risk	Low risk	Low risk	Low risk
Janik et al. (2021)	Some concerns	Low risk	Low risk	Low risk	Some concerns
